# 1709. Shifting Infection Spectrum in Febrile Neutropenic Pediatric Cancer Patients During the COVID-19 Pandemic: An Impact Assessment

**DOI:** 10.1093/ofid/ofad500.1542

**Published:** 2023-11-27

**Authors:** Aditya Dabas, Vagisha Sharma, Amitabh Singh, Kushal Kriplani, Rajni Gaind, Rani Gera

**Affiliations:** Vardhman Mahavir Medical College and Safdarjung Hospital, New Delhi, Delhi, India; Vardhman Mahavir Medical College and Safdarjung Hospital, New Delhi, Delhi, India; Vardhman Mahavir Medical College and Safdarjung Hospital, New Delhi, Delhi, India; Vardhman Mahavir Medical College and Safdarjung Hospital, New Delhi, Delhi, India; Vardhman Mahavir Medical College and Safdarjung Hospital, New Delhi, Delhi, India; Vardhman Mahavir Medical College and Safdarjung Hospital, New Delhi, Delhi, India

## Abstract

**Background:**

Febrile neutropenia (FN), which is the most common reason for hospital admission among pediatric oncology patients recieving chemotherapy, has seen changes in the epidemiology of infectious pathogens over the past few decades. This is attributable to shifts in hospital flora, antibiotics usage, and resistance patterns. Previously, bacterial infections were more common in this population, with mycobacterial, viral, and protozoal organisms being less frequent. Gram-negative bacteria such as E. coli, Klebsiella, and Pseudomonas were the most common causative agents. However, with the onset of the COVID-19 pandemic, the implementation of nationwide lockdowns, reduced healthcare visits, and strict interpersonal preventive measures are anticipated to impact the previous trends.

**Methods:**

A 16-month prospective observational study was conducted on 131 eligible pediatric cancer patients (ages 1-12) admitted with febrile neutropenia from Dec 2019 to May 2021. Causative pathogens were identified through lab investigations upon admission, and on days 3 and 7.

**Results:**

Out of the 131 children enrolled in the study, an etiological agent could be identified in 72. SARS-CoV-2, Acinetobacter, and Klebsiella accounted for over 50% of the infections. SARS-CoV-2 was the most common pathogen, and non-SARS-CoV-2 coronaviruses were also detected, even more than E. coli.Overall, gram-negative infectious agents comprised the most common pathogens (35.2%), similar to the pre-COVID era, followed by an appreciably increased respiratory viral burden (32.3%), a markedly decreased proportion of gram-positive infections (11.2%), and lastly, a largely unchanged fungal contribution (11.2%).

**Figure:**
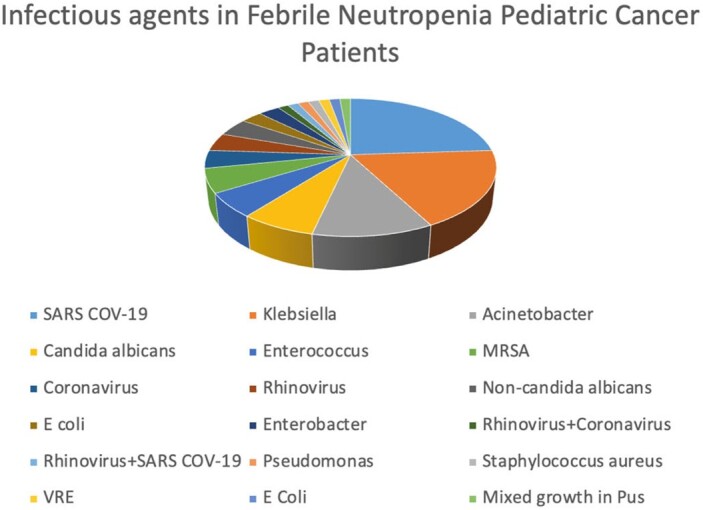

Major etiological agents in febrile neutropenia patients

**Conclusion:**

Rigorous interpersonal precautions and hygiene practices that were reinforced during the pandemic are effective in preventing hospital-acquired and contact-transmitted infections caused by gram-positive bacteria. However, despite the strict preventive measures and travel restrictions, transmission of airborne infections between immunocompromised patients and their caregivers was not avoided and became the most prevalent infection in the group. The risk of infection from the overgrowth of endogenous gram-negative flora remains unchanged.

**Disclosures:**

**All Authors**: No reported disclosures

